# Strain and sex dependent effects of isolation housing relative to environmental enrichment on operant sensation seeking in mice

**DOI:** 10.1038/s41598-021-97252-0

**Published:** 2021-09-08

**Authors:** Price E. Dickson, Guy Mittleman

**Affiliations:** 1 Department of Biomedical Sciences, Joan C. Edwards School of Medicine, Marshall University, 1700 3rd Ave., Huntington, WV 25703 USA; 2 Department of Psychological Science, Ball State University, North Quad (NQ), room 104, Muncie, IN 47306 USA; 3 Department of Psychology, University of Memphis, 400 Innovation Drive, Memphis, TN 38111 USA

**Keywords:** Epigenetics and behaviour, Reward

## Abstract

Sensation seeking is a multidimensional phenotype that predicts the development of drug addiction in humans and addiction-like drug seeking in rodents. Several lines of evidence suggest that chronic stress increases sensation seeking and addiction-like drug seeking through common genetic mechanisms. Discovery and characterization of these mechanisms would reveal how chronic stress interacts with the genome to influence sensation seeking and how drugs of abuse hijack these fundamental reward mechanisms to drive addiction. To this end, we tested the hypothesis that chronic isolation housing stress (relative to environmental enrichment) influences operant sensation seeking as a function of strain, sex, or their interaction. To determine if the BXD recombinant inbred panel could be used to identify genetic and epigenetic mechanisms underlying any identified gene-by-environment interactions, we used mice from the two BXD founder strains. Following 10 weeks of differential housing, we assessed operant sensation seeking using several reinforcement schedules. The primary finding from this study was that DBA/2J but not C57BL/6J mice were significantly vulnerable to an isolation-induced increase (relative to environmental enrichment) in sensation seeking during extinction when the sensory reward was no longer available; this effect was significantly more robust in females. These data reveal a previously unknown isolation-induced effect on extinction of operant sensation seeking that is sex-dependent, addiction-relevant, and that can be dissected using the BXD recombinant inbred panel.

## Introduction

Sensation seeking is a multidimensional phenotype that predicts the development of drug addiction in humans^1^. In mice and rats, genetic and phenotypic correlations among addiction-like drug use and sensation seeking indicate that these relationships are partially genetically driven^2–9^; selected line studies in rats also support this hypothesis^10^. Moreover, using the novelty reactivity model, environmental stress strain-dependently increases sensation seeking^11–14^ and drug use in rodents^15,16^; this suggests that stress influences biological mechanisms common to sensation seeking and addiction. Collectively, these data reveal the existence of shared genetic mechanisms driving addiction and sensation seeking; the genes and gene networks underlying these relationships remain unknown. Discovery and characterization of these shared genetic and epigenetic mechanisms would reveal the fundamental biological drivers of sensation seeking, how environmental factors interact with the genome to influence sensation seeking, and how drugs of abuse hijack these fundamental reward mechanisms to drive drug addiction.

Long-term isolation housing is a preclinical model of chronic stress that potentiates multiple addiction-relevant phenotypes. In contrast, environmental enrichment attenuates these behaviors^17^. Because the genome of mice has evolved in an environment that includes all enrichment aspects used in environmental enrichment experiments (e.g., exercise, social interaction, ability to build nest, relatively larger space for exploration), we consider environmental enrichment as the baseline housing condition and isolation housing as a treatment condition. Consequently, we describe effects of housing manipulations in which isolation housing is compared to environmental enrichment as “isolation-induced effects” for the remainder of the paper. Notably, when using only the two most extreme environmental conditions (i.e., isolation housing versus environmental enrichment), one cannot dissociate the distinct effects of each environmental factor on a phenotype. However, by using two extreme conditions, one can efficiently determine if a gene-by-environment interaction exists. If a gene-by-environment interaction is identified, subsequent studies can dissociate unique main effects and interactions among the many aspects of environmental enrichment.

Using the two inbred founder strains of the BXD recombinant inbred panel (C57BL/6J and DBA/2J), we have recently shown that, relative to environmental enrichment, isolation housing potentiates novelty reactivity, novelty preference, and anxiety via distinct genetic mechanisms in mice^11^. One of these three behaviors, novelty reactivity, has been described as an index of sensation seeking^3,10^. Novelty reactivity, quantified as distance traveled in a novel open field, is attractive as a measure of sensation seeking for several reason: the open field is a common apparatus in behavioral laboratories, the quantification of novelty reactivity is high-throughput, and the open field apparatus is relatively easy to set up and employ. However, novelty reactivity may be confounded by variation in locomotion or hyperactivity. A complementary and less frequently used index of sensation seeking is the operant sensation seeking (OSS) assay. OSS leverages the reinforcing nature of visual, auditory, and tactile sensations in animals. Specifically, mice will press a lever in an operant conditioning chamber to self-administer these stimuli, and the combined presentation of visual, auditory, and tactile stimuli serves as a strong reinforcer^9,18–20^. A disadvantage of the OSS paradigm is that it is lower throughput relative to novelty reactivity and more difficult to set up and employ. However, the key advantage of the OSS paradigm is that, with the exception of the reinforcer and the absence of an intravenous jugular catheter, it is identical to the gold standard intravenous drug self-administration paradigm. Therefore, acquisition, maintenance, progressive ratio breakpoint, and extinction of sensory stimulus self-administration can all be quantified using OSS. This provides a method for direct comparison of the effects of variables such as strain, sex, and housing condition on self-administration of sensory stimuli and self-administration of drugs of abuse.

In the present study, we assessed the effects of housing condition, strain, sex, and the interaction among these factors on the self-administration of a sensory reward composed of visual, auditory, and tactile components using the OSS paradigm. We assessed the effects of these factors on acquisition and extinction of the OSS response, fixed ratio one (FR-1) responding, and progressive ratio (PR) breakpoint. Immediately following weaning, littermates were housed in either (1) a large cage with conspecifics and environmental enrichment or (2) a standard shoebox cage without conspecifics or environmental enrichment. Following 10 weeks of differential housing, mice were tested on the OSS paradigm. To assess the influence of mouse strain on this phenomenon, we used mice from the two inbred founder strains of the BXD recombinant inbred panel (C57BL/6J and DBA/2J). The BXD panel was created by outcrossing mice from the C57BL/6J and DBA/2J progenitor strains and, following that, deriving new inbred strains from those offspring through sibling mating for ≥ 20 generations^21,22^. Consequently, the genometype of individual BXD strains consists of a unique and random combination of C57BL/6J and DBA/2J alleles. Therefore, at any genomic marker, individual BXD strains can be grouped into those that inherited the C57BL/6J allele at that marker or those that inherited the DBA/2J allele. Allele at a marker can then be used as the independent variable, and one can test the hypothesis that inherited allele at a marker accounts for significant variation on a phenotype. This approach, known as quantitative trait locus mapping^23^, has been used to identify gene candidates associated with behavioral and molecular phenotypes, including intravenous cocaine self-administration^6^ and operant sensation seeking^9^.

By performing this study, we hoped to answer three questions. First, does isolation housing (relative to environmental enrichment) influence sensation seeking. Second, if so, is this effect dependent on strain, sex, or both. Third, could the BXD recombinant inbred panel be used to discover the genetic and epigenetic mechanisms underlying such an effect. In this regard, because the C57BL/6J and DBA/2J inbred strains are the founders of the BXD panel, a significant interaction of strain and housing condition in the present study would confirm this. Collectively, answering these questions would confirm or exclude the possibility of using the OSS paradigm and the BXD mouse panel to discover the genetic and epigenetic mechanisms underlying the effects of stress on sensation seeking.

## Materials and methods

### Subjects and housing conditions

Experiments were conducted in The Department of Psychology at The University of Memphis and approved by the Institutional Animal Care and Use Committee at the University of Memphis. Experiments were conducted in accordance with the National Institutes of Health Guidelines for the Care and Use of Laboratory Animals and with the ARRIVE guidelines. Efforts were made to reduce the number of animals used and to minimize animal pain and discomfort.

The mouse strains, breeding protocol, and housing protocol used in the present study have been described in detail previously^11^. Briefly, offspring from male and female C57BL/6J mice (JAX stock number: 000664) and DBA/2J mice (JAX stock number: 000671) were weaned at 4 weeks of age and were used as experimental subjects. Following weaning, two mice of the same sex were randomly selected from a litter and those mice were randomly assigned to the isolation housing condition or environmental enrichment condition. Mice in the environmental enrichment condition were housed in same-sex groups in clear polycarbonate standard size rat cages that contained a vertical and horizontal running wheel for exercise, an opaque PVC tube and one half of a glove box for shelter, and three Nestlets for nest building. Mice in the isolation condition were housed individually in clear polycarbonate standard size mouse cages with no enrichment items. Mice were housed in these conditions for 10 weeks before being tested on the OSS paradigm. Mice remained housed in isolation or environmental enrichment conditions on the days that they were tested apart from the brief time that they were in the testing apparatus. Mice were maintained in a temperature-controlled environment (21 ± 1 °C) on a 12:12 light:dark cycle (lights on at 0800). Mice had free access to food and water throughout the experiment with the exception of the brief time in the testing apparatus. Prior to testing on the OSS paradigm, mice were tested for three days on a high throughput behavioral battery that consisted of novelty reactivity, novelty preference, and anxiety; these data have been reported separately^11^.

### Apparatus

The equipment used in the present study has been described in detail previously^18^. Briefly, OSS data were collected using Med Associates operant conditioning chambers enclosed in sound attenuating cubicles. Two retractable response levers were mounted on the front wall of each chamber and a stimulus light was mounted above each lever. A house light was centrally mounted on the rear wall of each chamber. Operant conditioning chambers were controlled by Lafayette Instruments (Lafayette, IN) BNC MK I control units.

### OSS testing

**Figure 1:**
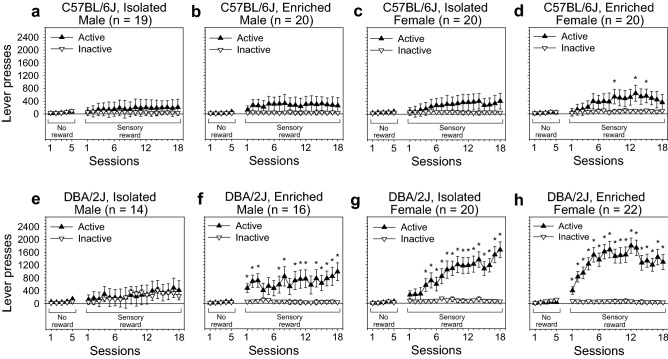
OSS acquisition curves for male and female C57BL/6J and DBA/2J mice that were housed long-term in one of two housing conditions: isolation or environmental enrichment. **(a**–**d)** OSS acquisition curves for male and female C57BL/6J mice in the isolation and enrichment conditions. **(e**,**f)** OSS acquisition curves for male and female DBA/2J mice in the isolation and enrichment conditions. *p < .05.

**Figure 2:**
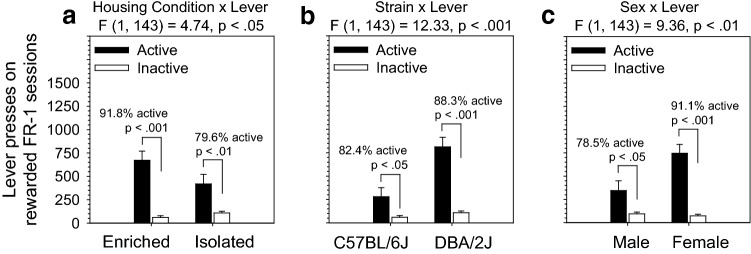
OSS on an FR-1 schedule was significantly influenced by housing condition, strain, and sex in C57BL/6J and DBA/2J mice. Across 18 FR-1 sessions, mice exhibited a significant preference for the active lever (*p* < .001), and this effect was significantly influenced by housing condition, strain, and sex. (**a**) The Housing × Lever interaction was driven by a significantly stronger dissociation between the active lever and inactive lever in enriched relative to isolated mice. The pattern of lever pressing was such that enriched mice had a stronger preference for the active lever relative to isolated mice. (**b**) The Strain × Lever interaction was driven by the production of a significantly greater number of active but not inactive lever presses by DBA/2J mice relative to C57BL/6J mice. (**c**) The Sex × Lever interaction was driven by the production of a significantly greater number of active but not inactive lever presses in female mice relative to male mice. Notably, neither strain nor sex interacted significantly with housing condition on the FR-1 schedule.

**Figure 3:**
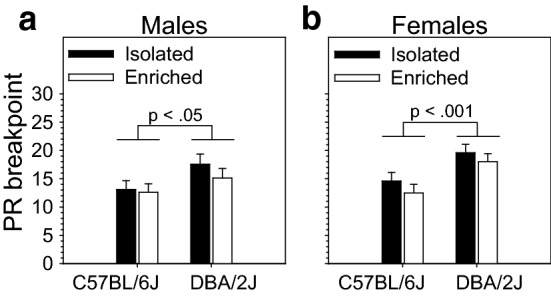
PR breakpoint on the OSS paradigm was significantly influenced by strain but not housing condition or sex in C57BL/6J and DBA/2J mice. (a, b) PR breakpoint for sensory stimuli-reinforced lever pressing was significantly influenced by strain (p < .001) but not sex or housing condition. DBA/2J mice reached a significantly higher breakpoint than C57BL/6J mice. This was true for both males and females.

**Figure 4:**
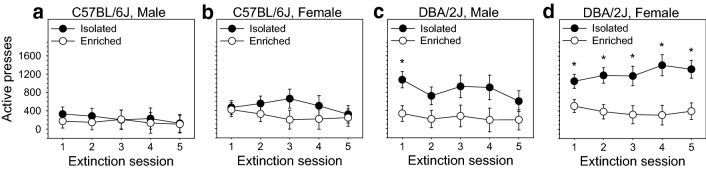
Extinction of the OSS response was significantly influenced by housing condition, and this relationship was significantly dependent on strain and sex. (**a**,**b**) There was no observed effect of housing on extinction responding in C57BL/6J mice, and this was true in both males and females. (**c**,**d**) In contrast, there was a robust and statistically significant effect of housing condition in DBA/2J mice: isolation housed male and female DBA/2J mice produced significantly more active but not inactive lever presses on the first extinction session relative to their environmentally enriched littermates. In female but not male DBA/2J mice, the number of active lever presses produced by isolation housed mice was significantly greater than the number produced by environmentally enriched mice throughout the remainder of the extinction sessions.

Mice were tested on four OSS stages during which distinct reward schedules were used: unrewarded lever pressing (Fig. 1), FR-1 (Figs. 1, 2), PR (Fig. 3), and extinction (Fig. 4). Acquisition of the OSS response was assessed using data from the FR-1 stage (Table 1). Testing occurred once per day, at the same time, seven days per week.

**Table 1:** The number of mice in each experimental group that acquired or failed to acquire the OSS response.

Strain/Sex/Housing	Acquisition statistics		
	Acquired	Failed	Percentage acquired
**C57BL/6J Male**			
Isolated	19	7	73.1%
Enriched	20	6	76.9%
**C57BL/6J Female**			
Isolated	20	5	80.0%
Enriched	20	5	80.0%
**DBA/2J Male**			
Isolated	14	7	66.7%
Enriched	16	5	76.2%
**DBA/2J Female**			
Isolated	20	6	76.9%
Enriched	22	4	84.6%
Total	151	45	77.0%

Mice met acquisition criteria if they performed ≥ 10 active lever presses with ≥ 70% of responses on the active lever for three consecutive sessions during the FR-1 stage. FR-1, progressive ratio, and extinction responding of mice that met acquisition criteria is shown in Figs. 1 – 4.

#### Unrewarded lever pressing

Unrewarded lever pressing sessions lasted for 75 min and began with the illumination of the house light and extension of the two response levers. Active and inactive lever presses were recorded but were unrewarded.

#### FR-1

FR-1 sessions were identical to unrewarded lever pressing sessions with the exception that an active lever press resulted in the delivery of a sensory reward composed of visual, auditory, and tactile components. Active lever side was counterbalanced: the right lever was active for half of the mice in each housing/strain/sex subgroup, and the left lever was active for the other half. To provide the visual component of the reward, the house light was extinguished and the stimulus lights above the active and inactive levers were rapidly illuminated and extinguished (i.e., flashed). Flash duration (1, 2, 4, or 8 s) and frequency (5, 2.5, 1.25, or 0.625 Hz) were randomized independently for each reward. The house light was re-illuminated once the flashing of the stimulus lights was terminated. The auditory and tactile components of the reward were accomplished by retraction, followed by immediate extension, of both the active and inactive levers. Retraction occurred at the same time that stimulus light flashing began. FR-1 sessions included a 15-min unrewarded block in the middle of the 75-min session that was not used in the analysis of OSS on an FR-1 schedule. So that the unrewarded lever pressing stage described in the section above and the FR-1 stage were directly comparable (Fig. 1), the 15-min block in the middle of the unrewarded lever pressing stage was excluded from analysis. Excluding the unrewarded block had no effect on the significance of main effects or interactions (Fig. 1 vs Fig. S1). The purpose of the unrewarded block was to provide a brief timeout in the middle of the session during which mice were not exposed to sensory stimuli; the goal was to reduce satiety for sensory stimuli. Randomization of flash duration and flash frequency was also intended to reduce satiety for sensory stimuli.

#### PR

PR sessions were identical to FR-1 sessions with the following exceptions: PR sessions lasted for 180 min, no unrewarded blocks were included, and rewards were delivered on a PR schedule rather than an FR-1 schedule. Specifically, following a reward, the number of active lever presses required for the delivery of the subsequent reward was increased according to the following sequence: 1, 2, 4, 6, 9, 12, 15, 20, 25, 32, 40, 50, 62, 77, 95, 118, 145, 178, 219, 268, 328, 402, 492, 603, 737, 901, 1102, 1347, 1646, 2012, 2459, 3004, 3670, 4484, 5478, 6692, 8175, 9986, 12,198.

#### Extinction

Extinction sessions were identical to FR-1 sessions with the following exceptions: extinction sessions lasted for 180 min and no rewards were delivered. Active and inactive lever presses were recorded.

### Statistical methods

Factorial analysis of variance (ANOVA) was used to assess the effects of the independent variables (housing condition, strain, sex, lever, session) and the interactions among these variables on the dependent variable lever pressing. Housing condition (isolated, enriched), strain (C57BL/6J, DBA/2J), and sex (male, female) were between-subjects factors. Session and lever (active, inactive) were within-subjects factors. Normality of all measures was assessed by inspecting normal probability plots. The assumption of homogeneity of variance across groups and timepoints was assessed using Mauchly’s test of sphericity. The Huynh–Feldt correction was used when this assumption was violated. When performing multiple comparisons, Fisher's Least Significant Difference procedure was used.

## Results

### Attrition

In total, 102 littermate pairs were tested (N = 204). Three DBA/2J mice and one C57BL/6J mouse in the enrichment condition were removed from the study due to fighting. These mice and their littermate controls were excluded from statistical analyses. To ensure that the lever pressing variable used in our analyses represented sensation seeking, mice that did not acquire the operant response for the sensory reward were excluded from analyses. Specifically, to determine if mice learned to press the active lever for delivery of the sensory reward during the FR-1 stage, we used criteria similar to those that we and others have used to assess successful acquisition of intravenous drug self-administration^6,24^: ≥ 10 active lever presses and 70% of responses on the active lever for three consecutive sessions. Out of 196 mice, 45 failed to meet these criteria (Table 1). Chi-square test for independence did not reveal a relationship between any of the independent variables and the dichotomous variable “met acquisition criteria”. All subsequent analyses were restricted to the 151 mice that met acquisition criteria. Testing of two mice was mistakenly terminated before they had fully completed the extinction stage. Consequently, those mice could not be included in the repeated measures ANOVA used to analyze extinction data.

### Unreinforced lever pressing was not influenced by housing, strain, or sex

Prior to the FR-1 stage during which a sensory reward was delivered following each active lever press, mice were tested under identical conditions with the exception that an active lever press did not result in reward delivery. Because FR-1 sessions included a 15-min unrewarded block in the middle of the session that was not included in statistical analysis, the same 15-min block in the middle of the unrewarded lever pressing stage was excluded from statistical analysis so that the two stages could be directly compared (Fig. 1). The goal of the “unreinforced lever pressing” stage was to identify any baseline differences in lever pressing driven by housing condition, strain, or sex so that they would not be misinterpreted as differences in sensation seeking during the FR-1 stage. ANOVA did not reveal a statistically significant effect of strain, sex, housing condition or interactions among these variables on lever pressing across the five sessions of non-reinforced lever pressing (Fig. 1).

### OSS on an FR-1 schedule was influenced by housing condition, strain, and sex

Acquisition of sensory stimuli-reinforced lever pressing for each of the housing/strain/sex subgroups is shown in Fig. 1. Across 18 FR-1 sessions, mice exhibited a significant preference for the active lever [F (1, 143) = 44.78, *p* < 0.001], and this effect was significantly influenced by housing condition, strain, sex, and session. The Housing × Lever interaction [F (1, 143) = 4.74, *p* < 0.05] was driven by a significantly stronger dissociation between the active lever and inactive lever in enriched relative to isolated mice (Fig. 2a). The pattern of lever pressing was such that enriched mice had a stronger preference for the active lever relative to isolated mice. The Strain × Lever interaction [F (1, 143) = 12.33, *p* < 0.001] was driven by the production of a significantly greater (p < 0.001) number of active but not inactive lever presses by DBA/2J mice relative to C57BL/6J mice (Fig. 2b). The Sex × Lever interaction [F (1, 143) = 9.36, *p* < 0.01] was driven by the production of a significantly greater (*p* < 0.001) number of active but not inactive lever presses in female mice relative to male mice (Fig. 2c). The Session × Lever interaction [F (17, 2431) = 8.36, *p* < 0.001] was driven by a significant increase in preference for the active lever across sessions. Notably, neither strain, sex, nor session interacted significantly with housing condition on the FR-1 schedule. The main effects and interactions described in this paragraph were statistically significant when excluding the unrewarded block (Fig. 1) and when including the unrewarded block (Fig. S1). Active lever preference data are shown in Fig. S2. Individual data points for analyses depicted in Fig. 2 are shown in Fig. S3.

### PR breakpoint on the OSS paradigm was influenced by strain but not housing condition or sex

PR breakpoint on the OSS paradigm was significantly influenced by strain [F (1, 143) = 15.61, *p* < 0.001] but not housing condition or sex. DBA/2J mice reached a significantly higher breakpoint than C57BL/6J mice (Fig. 3). This was true for both males (Fig. 3a) and females (Fig. 3b). Individual data points are shown in Fig. S4.

### Extinction of the OSS response was strongly influenced by housing condition, and this relationship was significantly dependent on strain and sex

Extinction of sensory stimuli-reinforced lever pressing was significantly influenced by housing condition [F (1, 141) = 14.97, *p* < 0.001], strain [F (1, 141) = 13.01, *p* < 0.001], and sex [F (1, 141) = 9.23, *p* < 0.01]. Notably, the two-way interaction of strain and housing condition was statistically significant [F (1, 141) = 6.35, *p* < 0.05] and the three-way interaction of sex, housing condition, and session was statistically significant [F (4, 564) = 3.30, *p* < 0.05]. The main effect of session was not statistically significant [F (4, 564) = 1.43, *p* = 0.23]. Collectively, these analyses indicate that the effect of housing on extinction responding was significantly dependent on strain and sex. In this regard, there was no observed effect of housing on extinction responding in C57BL/6J mice, and this was true in both males (Fig. 4a) and females (Fig. 4b). In contrast, there was a robust and statistically significant effect of housing condition in DBA/2J mice: isolation housed male (Fig. 4c) and female (Fig. 4d) DBA/2J mice produced significantly more (*p* < 0.01 and *p* < 0.05, respectively) active lever presses on the first extinction session relative to their environmentally enriched littermates. Critical to the interpretation of this finding is the observation that inactive lever presses did not differ between isolation housed and environmentally enriched mice in any of the strain/sex subgroups on the first session. In female (Fig. 4d) but not male (Fig. 4c) DBA/2J mice, the number of active lever presses produced by isolation housed mice was significantly greater than the number produced by environmentally enriched mice throughout the remainder of the extinction sessions. Collectively, these data reveal a vulnerability to isolation housing (relative to environmental enrichment) on OSS extinction responding in DBA/2J mice, but not C57BL/6J mice, that is most robust in female mice.

## Discussion

In the present study, we assessed the effects of housing condition (environmental enrichment, isolation housing), mouse strain (C57BL/6J, DBA/2J), and sex on the OSS paradigm, an operant model of sensation seeking. The most robust effect of environmental enrichment was observed during the extinction stage, and this effect was significantly strain dependent and sex dependent. Specifically, isolation housed mice from the DBA/2J strain exhibited significantly higher extinction responding relative to environmentally enriched littermates (Fig. 4), and this effect was most robust in females. No effect of housing condition on extinction responding was observed in C57BL/6J mice. During the FR-1 stage (Fig. 2), environmentally enriched mice exhibited a stronger preference for the active lever relative to isolation housed mice; both DBA/2J mice and female mice (relative to C57BL/6J mice and male mice, respectively) exhibited significantly greater responding on and a stronger preference for the active lever on the FR-1 stage. During the progressive ratio stage, DBA/2J mice reached a significantly higher breakpoint than C57BL/6J mice irrespective of sex or housing condition. Collectively, these data provide novel observations of the effect of housing condition, strain, and sex on OSS and replicate several of our previous findings. Most importantly, these data reveal a novel and sex-dependent isolation-induced vulnerability (relative to environmental enrichment) to operant sensation seeking during extinction that is observed in DBA/2J but not C57BL/6J mice. Because C57BL/6J and DBA/2J are the founder strains of the biparental BXD recombinant inbred panel, the BXD panel can be used to discover the genetic and epigenetic mechanisms underlying this addiction-relevant gene-by-environment interaction.

### Long-term isolation housing (relative to environmental enrichment) sex-dependently increased sensation seeking during OSS extinction in DBA/2J but not C57BL/6J mice

Relative to environmental enrichment, isolation housing beginning at weaning and lasting through the remainder of the study significantly increased active lever pressing during OSS extinction in DBA/2J mice (Fig. 4). This effect was sex dependent in that it persisted through all five days of extinction in females but was significantly observed in males on only the first extinction session. Notably, the effect of housing condition on extinction responding observed in DBA/2J mice was completely absent in C57BL/6J mice. This observation reveals the existence of a sex-dependent vulnerability to long-term isolation housing (relative to environmental enrichment) in the DBA/2J but not C57BL/6J strain. This effect may be driven by isolation-induced changes to neuronal mechanisms underlying the extinction burst. It is also possible that this effect is influenced by changes to mechanisms influencing compulsivity, impulsivity, or the progression to habitual behavior.

Several points are relevant to the interpretation of this phenomenon. First, the observed effect in DBA/2J mice of housing condition on OSS extinction was not due to a more general effect on locomotion or exploration. We know this because on the first day of extinction (i.e., the day on which the housing-dependent effect was most robust), inactive lever presses did not differ between isolation housed and environmentally enriched mice. Second, the strain dependent effect of housing on extinction responding was not secondary to a previous strain dependent effect on FR-1 responding or PR breakpoint. We know this because a strain dependent effect of housing was not observed at either of those stages. One caveat to this is that exposure to the unrewarded block during the FR-1 stage may have influenced responding during the extinction stage. Collectively, these data indicate that the isolation induced vulnerability that was observed in DBA/2J mice is specific to the extinction stage and reflects volitional sensation seeking as opposed to generalized changes in locomotion or exploration.

Several lines of work suggests that the strain dependent effect of housing condition on OSS extinction observed in the present study is relevant to addiction. Specifically, previous studies in rats have shown that environmental enrichment reduces psychostimulant seeking and sucrose seeking during extinction relative to isolation housed controls^25,26^. Moreover, several studies have revealed that the biological mechanisms driving operant sensation seeking are shared with those driving drug and alcohol seeking^9,19,27,28^. Collectively, these data suggest that environmental enrichment reduces operant psychostimulant seeking and operant sensation seeking through shared biological mechanisms, and that genometype influences this effect. The underlying mechanisms driving this effect are currently unknown, but may involve effects on neurogenesis in hippocampus or other brain regions influencing reward^29,30^. Because the two strains used in the present study (C57BL/6J, DBA/2J) are the progenitor strains of the BXD recombinant inbred panel, the findings presented here indicate that a systems genetics study using the BXD strains could be used to reveal the genetic and epigenetic mechanisms underlying the isolation-induced vulnerability to reward seeking observed in the DBA/2J strain.

### Housing condition, strain, and sex independently influenced OSS

In the present study, we observed a main effect of housing condition, strain, and sex on OSS during the FR-1 stage. Notably, these variables did not interact. We describe each of these effects below.

Irrespective of strain or sex, environmental enrichment increased preference for the active lever relative to isolation housing on the FR-1 stage (Fig. 2a). Because lever pressing as a whole did not differ significantly between environmentally enriched and isolation housed mice (i.e., no main effect of housing), we interpret this phenomenon as reflecting a greater ability to dissociate between the two levers rather than a stronger preference for the reward itself. Consequently, the observed housing effect may be caused by facilitated learning in environmentally enriched mice. It is also possible that the ability to dissociate the two levers reflects reduced hyperactivity in environmentally enriched C57BL/6J and DBA/2J mice^11,31^. Reduced hyperactivity could lead to greater on-task performance and, consequently, reduced inactive and greater active lever pressing.

Irrespective of housing condition or sex, DBA/2J mice pressed the active but not inactive lever significantly more on the FR-1 stage than C57BL/6J mice (Fig. 2b). Secondary to this effect was an increased preference for the active lever in DBA/2J mice. We also observed that DBA/2J mice reached a significantly higher PR breakpoint relative to C57BL/6J mice (Fig. 3). The increased active lever pressing observed in DBA/2J mice is unlikely to be secondary to increased locomotion because of significantly lower open field locomotion in DBA/2J relative to C57BL/6J^11^. Our findings in this study replicate our findings from a previous study in which DBA/2J mice self-administered more sensory stimuli than C57BL/6J mice on an FR-1 schedule^18^. Collectively, our data from this study and our previous study investigating OSS in mice indicate that DBA/2J mice are higher sensation seekers on the OSS paradigm relative to C57BL/6J mice. One explanation for these findings is that DBA/2J mice have a relatively higher homeostatic set point of sensory stimulation^32^ in comparison to C57BL/6J mice. This is consistent with our findings of significantly higher novelty place preference in DBA/2J mice relative to C57BL/6J mice^11^.

Irrespective of housing condition and strain, female mice pressed the active but not inactive lever significantly more on the FR-1 stage than male mice (Fig. 2c). Consequently, relative to male mice, female mice exhibited a stronger preference for the active lever. On its own, this suggests higher sensation seeking in female mice rather than a reduced ability to dissociate the active from the inactive lever. However, on the PR stage, the breakpoint reached by female and male mice was equivalent (Fig. 3). Consequently, it is unclear whether female mice were exhibiting increased sensation seeking, an increased ability to dissociate the active from the inactive lever, or a combination of the two. Notably, in our previous study of operant sensation seeking in mice from the BXD RI panel^9^, we observed significantly higher sensation seeking in male relative to female mice. One explanation for this may be the significant strain by sex interaction observed in that study indicating that the existence of sex differences and the directionality of those differences is dependent on mouse strain. Thus, although sensation seeking was greater in female mice from some BXD strains in that study, the specific BXD strains tested resulted in overall greater sensation seeking in male mice.

Collectively, operant sensation seeking data from this experiment and our previous two operant sensation seeking experiments^9,18^ indicate the following. First, environmental enrichment facilitates learning in operant conditioning paradigms as indicated by greater lever dissociation in enriched mice. Second, relative to C57BL/6J mice, DBA/2J mice exhibit significantly greater sensation seeking in the OSS paradigm on both FR-1 and PR schedules. Third, relative to males, female C57BL/6J and DBA/2J mice exhibit facilitated learning, higher sensation seeking, or a combination of these two phenomena; this effect is influenced by genometype in the BXD RI panel.

### Systems genetics for discovery of genetic and epigenetic mechanisms driving isolation-induced vulnerability to addiction-like reward seeking

We conducted the present study in order to answer three questions. First, does isolation housing (relative to environmental enrichment) influence sensation seeking or extinction of the sensation seeking response. Second, if so, is this effect dependent on strain, sex, or both. Third, could the BXD recombinant inbred panel be used to discover the genetic and epigenetic mechanisms underlying such an effect. In this regard, we discovered a strain- and sex-dependent effect of isolation housing on extinction of the OSS response: DBA/2J mice were vulnerable to an isolation-induced increase in sensation seeking during extinction relative to environmentally enriched controls; this effect was significantly more robust in females. Critically, no effect of housing condition on extinction of the OSS response was observed in C57BL/6J mice. This finding reveals an epigenetic phenomenon driven by alleles in the DBA/2J mouse strain. Because “strain” was the independent variable in the present study, we could only determine that these alleles exist, not the identity of the alleles themselves. To identify the alleles underlying the observed effect, it will be necessary to use a design in which “allele” rather than “strain” is the independent variable. As described in the introduction section, the BXD panel allows for such a design.

In order to identify genes associated with the isolation-induced vulnerability identified in the present study, mice from multiple BXD strains would be exposed to differential housing conditions and then tested on the OSS paradigm as reported in the present study. By using allele as the independent variable, specific genomic regions accounting for variation on isolation-induced extinction of the OSS response could be identified. Moreover, by quantifying gene expression (RNA-seq) and chromatin accessibility (ATAC-seq) in differentially housed but behaviorally naïve mice from these same BXD strains, the genomic and epigenomic mechanisms associated with variation on isolation-induced extinction of the OSS response could be identified. Because sensation seeking and drug addiction are driven by shared biological mechanisms, identification of genetic mechanisms driving stress-induced vulnerability to sensation seeking may reveal mechanisms driving stress-induced vulnerability to drug addiction.

## Supplementary Information

Supplementary Figures.
