# Supplementary material for: Strain and sex dependent effects of isolation housing relative to environmental enrichment on operant sensation seeking in mice
Source: Sci Rep. 2021 Sep 8;11:17826. doi: 10.1038/s41598-021-97252-0 (PMC8426458; doi:10.1038/s41598-021-97252-0)
Supplement: Supplementary file 1 — Supplementary Figures. [file 41598_2021_97252_MOESM1_ESM.pdf]

Strain and sex dependent effects of isolation housing relative to environmental enrichment on operant sensation seeking in mice

Price E. Dickson[*1, 3] and Guy Mittleman[2, 3]

1. Department of Biomedical Sciences
   Joan C. Edwards School of Medicine
   Marshall University
   1700 3rd Ave.
   Huntington, WV 25703

2. Department of Psychological Science
   North Quad (NQ), room 104
   Ball State University
   Muncie, IN 47306

3. Department of Psychology
   University of Memphis
   400 Innovation Drive
   Memphis, TN 38111

* Corresponding author

Price E. Dickson, Ph.D.

price.dickson@marshall.edu

Supplementary figures and captions

Figure S1. FR-1 analysis including the unrewarded block. We analyzed FR-1 data in two ways. In the first which is shown in Figure 1, we excluded lever pressing from an unrewarded 15-minute block which was placed in the middle of the 75-minute session. The purpose of designing the study with the unrewarded block was to reduce sensory stimulus satiety. In the second analysis which is shown in this supplementary figure, we included active and inactive lever presses which occurred during the 15-minute unrewarded block. The binary outcome of the statistical tests (i.e., statistically significant or not statistically significant) was equivalent in both analyses and the F statistics were similar. The F tests for the analysis in which we included the unrewarded block are shown below.

- Housing x Lever interaction [$F(1, 143) = 4.12$, $p < .05$]
- Strain x Lever interaction [$F(1, 143) = 11.91$, $p < .001$
- Sex x Lever interaction [$F(1, 143) = 9.48$, $p < .01$]
- Session x Lever interaction [$F(17, 2431) = 8.57$, $p < .001$]

Figure S2. Active lever preference during FR-1 sessions. Thin lines represent individual mice. Black circles with error bars represent the mean and standard error of all mice in the panel.

Figure S3. OSS on an FR-1 schedule. Individual data points (collapsed on session) for the analyses displayed in Figure 2.

Figure S4. OSS on a PR schedule. Individual data points for the analysis of PR data displayed in Figure 3.

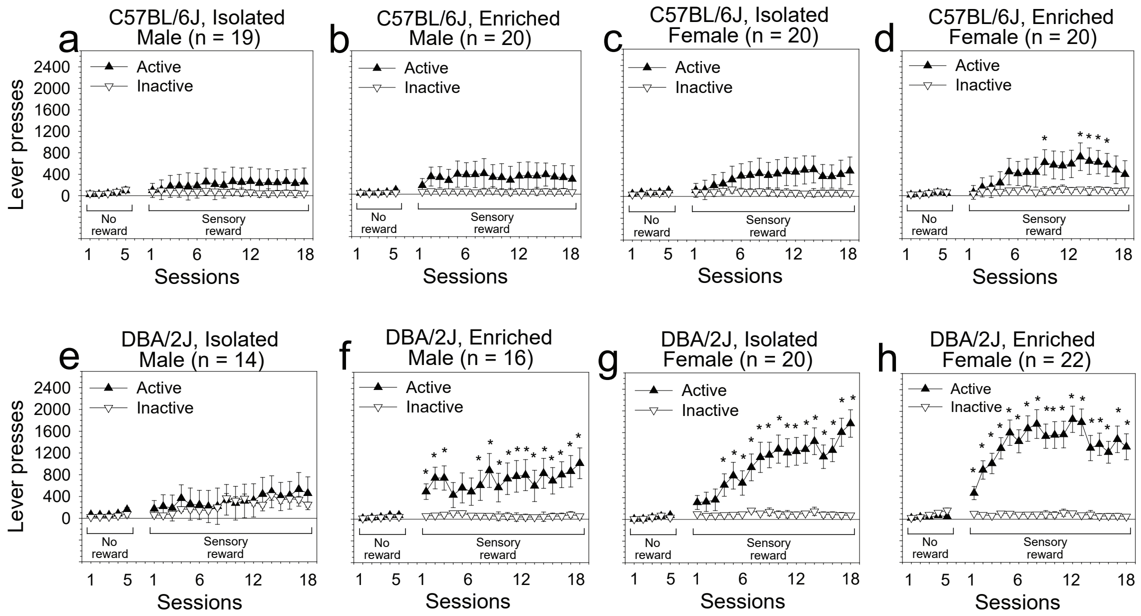

a
C57BL/6J, Isolated
Male (n = 19)
b
C57BL/6J, Enriched
Male (n = 20)
c
C57BL/6J, Isolated
Female (n = 20)
d
C57BL/6J, Enriched
Female (n = 20)
e
DBA/2J, Isolated
Male (n = 14)
f
DBA/2J, Enriched
Male (n = 16)
g
DBA/2J, Isolated
Female (n = 20)
h
DBA/2J, Enriched
Female (n = 22)
Active
Inactive
Lever presses
No reward
Sensory reward
Sessions

Figure S1.

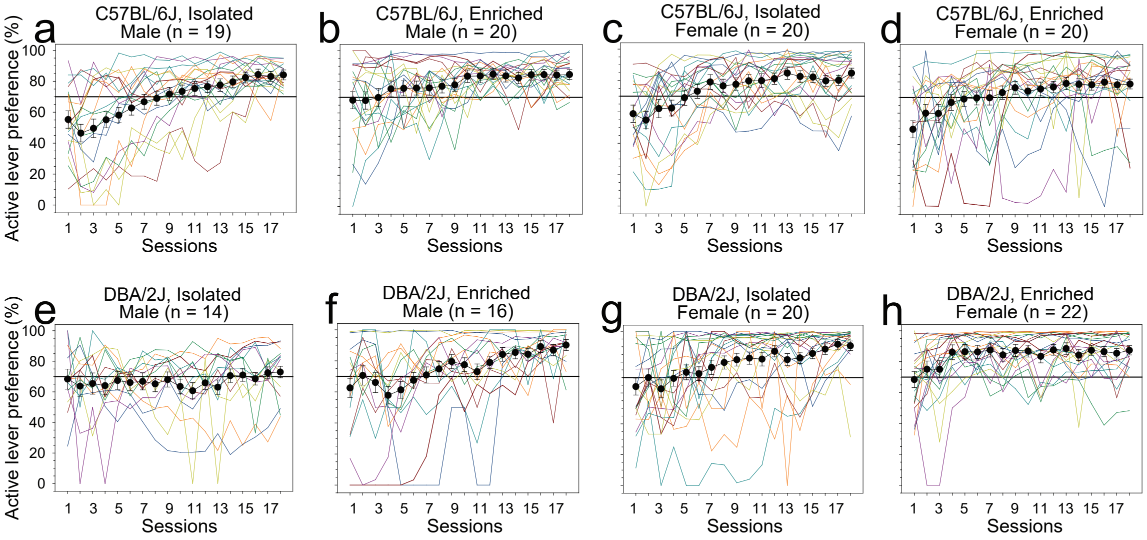
a
C57BL/6J, Isolated
Male (n = 19)
b
C57BL/6J, Enriched
Male (n = 20)
c
C57BL/6J, Isolated
Female (n = 20)
d
C57BL/6J, Enriched
Female (n = 20)
e
DBA/2J, Isolated
Male (n = 14)
f
DBA/2J, Enriched
Male (n = 16)
g
DBA/2J, Isolated
Female (n = 20)
h
DBA/2J, Enriched
Female (n = 22)
Active lever preference (%)
Sessions

Figure S2.

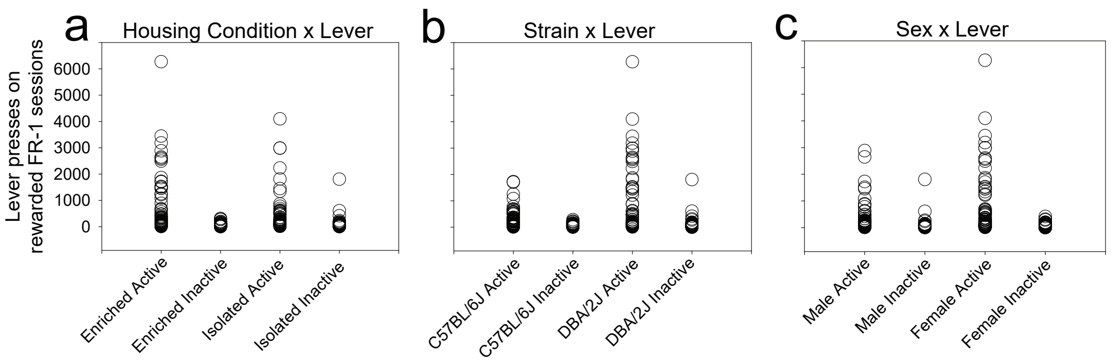
a
Housing Condition x Lever
Lever presses on rewarded FR-1 sessions
6000
5000
4000
3000
2000
1000
0
Enriched Active
Enriched Inactive
Isolated Active
Isolated Inactive
b
Strain x Lever
C57BL/6J Active
C57BL/6J Inactive
DBA/2J Active
DBA/2J Inactive
c
Sex x Lever
Male Active
Male Inactive
Female Active
Female Inactive

Figure S3.

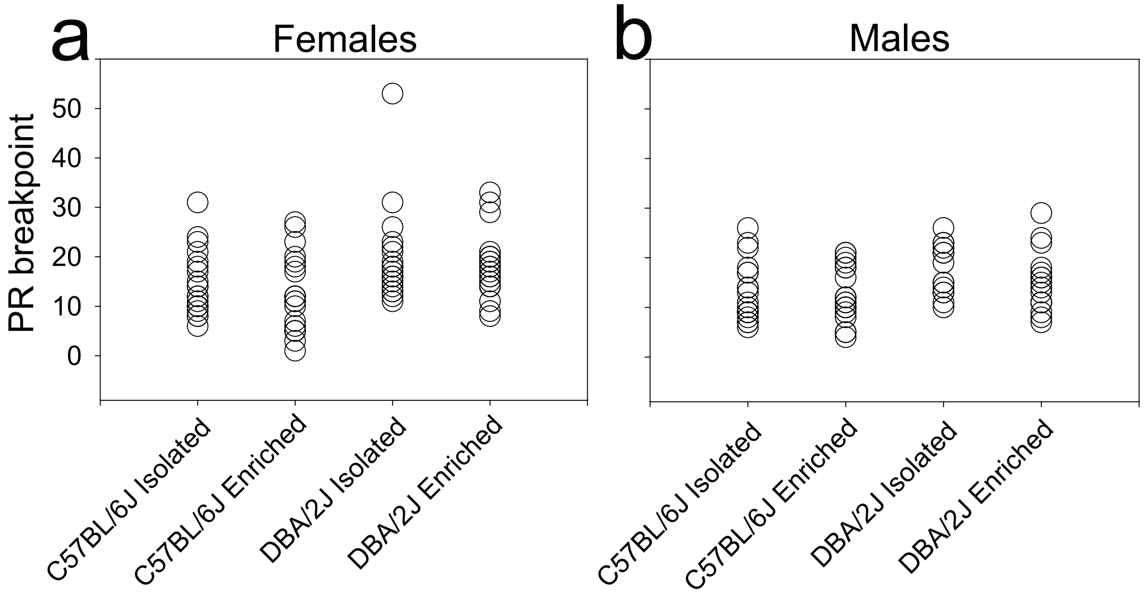
a
Females
b
Males
PR breakpoint
0
10
20
30
40
50
C57BL/6J Isolated
C57BL/6J Enriched
DBA/2J Isolated
DBA/2J Enriched

Figure S4.
